# BATCH-GE: Batch analysis of Next-Generation Sequencing data for genome editing assessment

**DOI:** 10.1038/srep30330

**Published:** 2016-07-27

**Authors:** Annekatrien Boel, Woutert Steyaert, Nina De Rocker, Björn Menten, Bert Callewaert, Anne De Paepe, Paul Coucke, Andy Willaert

**Affiliations:** 1 Center for Medical Genetics, Ghent University Hospital, Ghent, Belgium

## Abstract

Targeted mutagenesis by the CRISPR/Cas9 system is currently revolutionizing genetics. The ease of this technique has enabled genome engineering *in-vitro* and in a range of model organisms and has pushed experimental dimensions to unprecedented proportions. Due to its tremendous progress in terms of speed, read length, throughput and cost, Next-Generation Sequencing (NGS) has been increasingly used for the analysis of CRISPR/Cas9 genome editing experiments. However, the current tools for genome editing assessment lack flexibility and fall short in the analysis of large amounts of NGS data. Therefore, we designed BATCH-GE, an easy-to-use bioinformatics tool for batch analysis of NGS-generated genome editing data, available from https://github.com/WouterSteyaert/BATCH-GE.git. BATCH-GE detects and reports indel mutations and other precise genome editing events and calculates the corresponding mutagenesis efficiencies for a large number of samples in parallel. Furthermore, this new tool provides flexibility by allowing the user to adapt a number of input variables. The performance of BATCH-GE was evaluated in two genome editing experiments, aiming to generate knock-out and knock-in zebrafish mutants. This tool will not only contribute to the evaluation of CRISPR/Cas9-based experiments, but will be of use in any genome editing experiment and has the ability to analyze data from every organism with a sequenced genome.

Research on gene function and human genetic disorders has taken immense steps forward by the use of animal model systems, which can be generated by a number of genetic tools, ranging from random ENU mutagenesis to the recently emerged target-specific genome editing techniques, such as Zinc Finger Nucleases (ZFNs)^1^,^2^, Transcription Activator–Like Effector Nucleases (TALENs)^3^,^4^ and type II Clustered Regularly Interspaced Short Palindromic Repeats/CRISPR-Associated Systems (CRISPR/Cas9)^5^,^6^,^7^. In particular, CRISPR/Cas9 is rapidly evolving into the method of choice due to its simplicity and high efficiency of mutagenesis.

The CRISPR/Cas9 technique is derived from a bacterial and archaeal defensive system, providing adaptive immunity against invading viruses and plasmids^8^,^9^,^10^. After uptake of foreign nucleic acids, these organisms can integrate a short fragment of this foreign DNA, which is called the protospacer, into the clustered regularly interspaced short palindromic repeat (CRISPR) locus of the host. These protospacer sequences are selected by the proximity of a short sequence, the protospacer adjacent motif (PAM). CRISPR locus transcription and processing leads to the production of the CRISPR RNA (crRNA) that forms a complex with a trans-activating crRNA (tracrRNA). This complex guides Cas9, a RNA-guided endonuclease (RGEN), transcribed from the host’s CRISPR-associated (Cas) locus, to the target sequence. Cas9 only exerts its DNA cleavage function if the target DNA sequence contains a PAM.

A range of Cas9 variants exists, all of which possess different PAM dependencies. Functioning of Cas9 RGEN from *Streptococcus pyogenes*, for instance, relies on the presence of a 5′-NGG-3′ PAM, located immediately downstream of the protospacer^6^. Cas9 endonuclease activity introduces double strand breaks (DSB), approximately 3 base pairs (bp) upstream of the PAM motif. In response to DSB generation, two endogenous DNA repair mechanisms can be initiated: non-homologous end joining (NHEJ) and homology-directed repair (HDR)^11^. NHEJ is an error-prone repair mechanism, which can lead to small base pair insertions and deletions (indels). HDR on the other hand, relies on the presence of a homologous piece of donor DNA to repair the DNA break and will more likely repair the DSB correctly^12^.

The CRISPR/Cas9 bacterial defence mechanism has been modified into a versatile and easily applicable system, which solely relies on the combined use of a customizable target-specific single-guide RNA (sgRNA) and its accompanying Cas9 protein^6^. This approach has been increasingly and successfully applied in a range of organisms to achieve either gene knock-out or knock-in. The outcome of these genome editing experiments can be assessed by a number of methods. On the one hand, mutagenesis efficiency has generally been estimated by enzymatic mutation detection techniques, of which the T7 endonuclease I (T7E1)^13^ and the Surveyor’s assay^14^ are most well-known. The principal drawback of these techniques is their limited mutation detection sensitivity. Minimal sensitivities of 5 to 10% have been reported^15^. In addition, these enzymatic techniques have restrictions regarding the detection of specific types of mutations. The Surveyor’s assay is less suitable for the detection of indel mutations, whereas T7E1 is inappropriate for identifying transition and transversion mutations^15^,^16^,^17^. Moreover, these techniques are not able to reveal the specific sequence alterations in genome editing experiments. This can be achieved by cloning of PCR-amplified target regions, followed by Sanger sequencing. However, this approach is labour-intensive and generates too little data to reliably determine the full range of mutations and their frequencies in a specific sample. The aforementioned drawbacks can be overcome by the use of the Next-Generation Sequencing (NGS) technology. NGS has made tremendous progress in terms of speed, read length, throughput and cost and has become available for most research institutions^18^. With NGS, thousands to millions of sequencing reactions can be performed in parallel and thus a valuable amount of information is generated. However, the processing of raw sequencing data into a clear report that assesses the mutation efficiency of different types of mutations in a genome editing experiment is not straightforward. The NGS output can be simply explored in a visualization tool, such as The Integrative Genomics Viewer (IGV)^19^,^20^, although this tool does not provide any further analysis modalities. The data can be analysed more in-depth using the CRISPR Genome Analyzer platform (CRISPR-GA), currently the only bio-informatics tool that provides information on size and location of indels and on the efficiency of NHEJ and HDR events^21^. CRISPR-GA is limited to a one-by-one sample analysis, which is not time-efficient when dealing with large sample sets. We therefore developed BATCH-GE, a tool that provides a more detailed analysis of genome editing experiments and supports batch analysis of multiple samples. The tool is implemented as a freely available Perl script and can be run on any Linux-based server or personal computer, ensuring easy accessibility. The generation of a variant table providing a detailed overview of the type, chromosomal position, length and frequency of the generated indel mutations allows a thorough evaluation of gene editing experiments. In addition, BATCH-GE enables assessment of HDR-mediated precise genome editing experiments by returning efficiencies for one or multiple intended base pair substitutions. Multiple genome editing experiments can be analysed in a batchwise manner with limited and simple input requirements. BATCH-GE does not only aid in the analysis of CRISPR/Cas9-based experiments, but can assess the outcome of any genome editing experiment.

## Results

### BATCH-GE implementation

**Figure 1:**
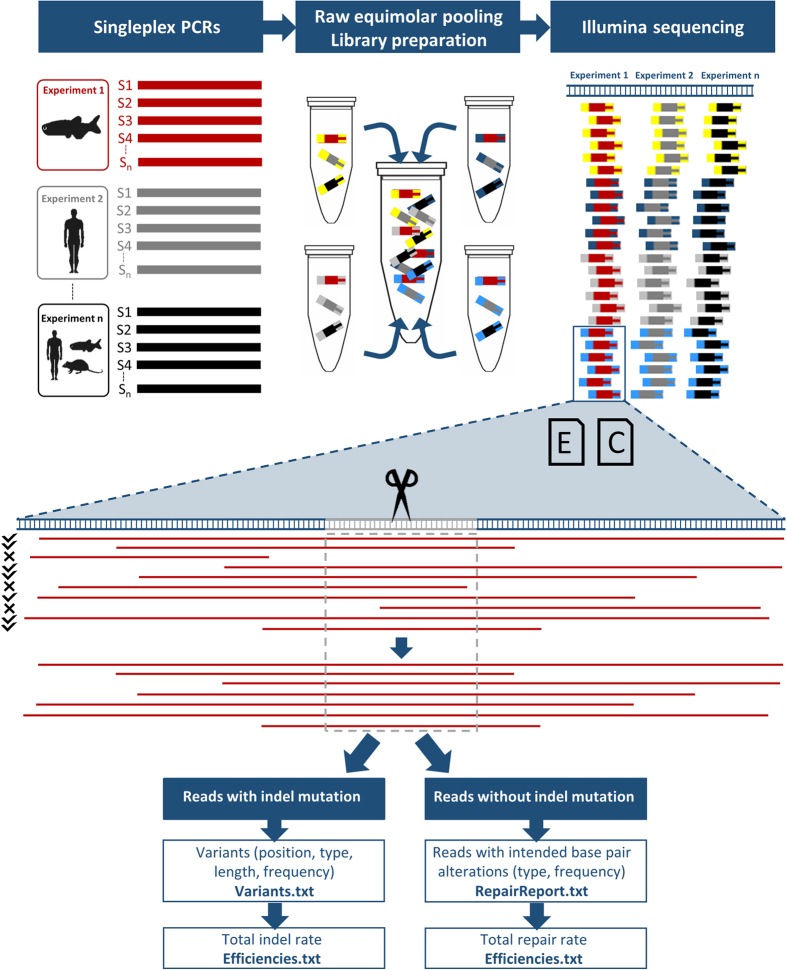
Implementation of BATCH-GE. Multiple singleplex PCR products (S1, S2, …, Sn) (upper panel, left) that correspond to different genomic sequences in one specific or in different genomes are pooled in equimolar amounts. Subsequently, the pools are used as DNA input for NGS library preparation using the Nextera XT library preparation kit, which simultaneously fragments and tags input DNA (upper panel, middle). The tagging involves the addition of unique adapter sequences in order to provide sequencing indices on both sides of the amplicons (depicted by yellow, grey, light and dark blue bars). In a final step, all molecules are pooled in a single tube prior to NGS sequencing (upper panel, right). BATCH-GE analyses the data sample-by-sample in an automated batchwise manner. The experimental specifications needed to run BATCH-GE are supplied via two input files (middle panel, E (Experiment.csv) and C (Cutsites.bed) icons). In a first step, raw sequencing data is converted into the SAM file format. Secondly, BATCH-GE screens the reads in the SAM file for their coverage of the region(s) of interest, which are user-defined regions, encompassing the theoretical CRISPR/Cas9 cut site, 3 base pairs upstream of the PAM sequence (middle panel, grey sequence). Thirdly, reads that do not fully cover the region of interest are discarded from the analysis, since they lack information about the presence or absence of indels in this region (middle panel, indicated by a mark/cross). Subsequently, the remaining reads (indicated by a tick) are screened for insertions and deletions initiated within the same user-defined region of interest (middle panel, grey dash-lined box). The detected indel variants, along with information about their position, type, length and their frequency are written to a ‘Variants’ text file. Reads that do not contain any indel, are screened for the presence of intended base pair alterations. Frequencies of partial and full repairs are listed in the ‘RepairReport’ file. Additionally, general indel and repair rates are indicated in the ‘Efficiencies’ file. Lastly, URLs (‘URL’ file) enable read visualization in the freeware UCSC Genome Browser database^22^.

The BATCH-GE analysis tool requires NGS-derived sequencing data (Fig. 1). As a first step, DNA derived from a genome editing experiment is singleplex PCR-amplified for the corresponding region of interest. Multiple PCR products covering different genomic regions in one specific or different genomes can be pooled together for DNA library preparation and NGS. Following NGS, BATCH-GE directly uses raw NGS data (fastQ file format) as input to generate a detailed report on genome editing efficiencies. Analysis is carried out sample-by-sample in an automated manner, where the user only needs to provide two input files: a comma delimited (csv) experiment file (Table 1, Supplementary Fig. S1) and a BED file (Table 2, Supplementary Fig. S1) in which the cut sites are specified. In the 4-column BED-file, the first 3 columns define the genomic coordinates for the user-defined region of interest comprising the particular cut site and the 4^th^ column contains the identifier of the cut site as defined in the experiment file. The experiment file contains all information that is specific for a particular experiment, i.e. the full path of the directory of the FastQ files, an identifier of the sample, the genome that should be used for mapping, an identifier of the CRISPR/Cas9 cut site which is mostly the name of a gene or other genomic element, the path to the directory where the results should be written, the path to the second input file, the ‘Cutsites.bed’ file where the genomic coordinates of the region of interest can be found and optionally an HDR template sequence. The experiment file can contain any number of samples from different sequencing runs and covering various regions of interest in different genomes.

**Figure 2:**
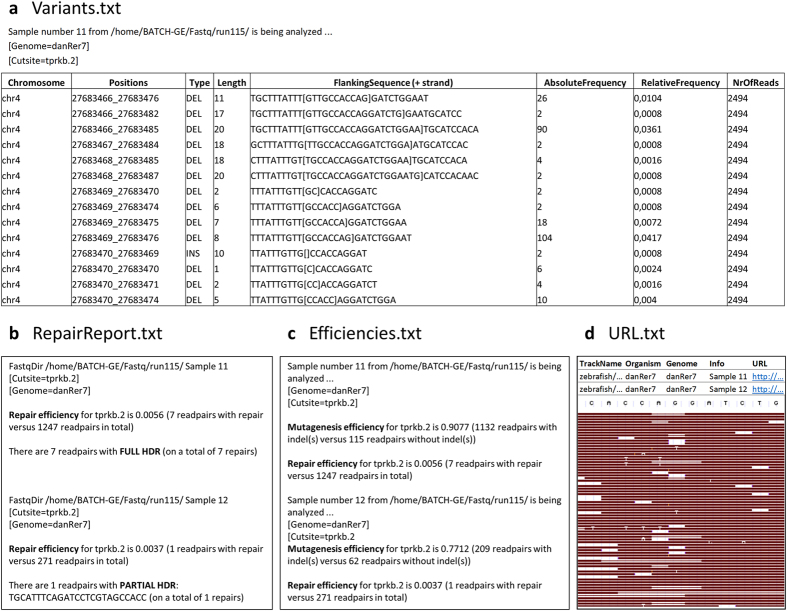
BATCH-GE output files for a specific genome editing experiment targeting the *tprkb* gene. (**a**) The ‘Variants’ text file lists chromosome, chromosomal location of the variant, type of the variant, length, the reference sequence surrounding the indel (10 bp upstream and 10 bp downstream of the indel) with [] marking the inserted sequence or with [*deleted base pairs*] marking the deleted sequence, and absolute and relative frequency of the variants. **(b)** In case of HDR analysis, the reads which do not contain any indel, are screened for the presence of the intended base pair alterations. BATCH-GE can distinguish between full and partial repair, in case multiple base pair alterations are intended to be introduced in the region of interest. If partial repair is encountered, the specific sequence of the partial repair is listed. **(c)** General indel and repair rates are shown in the ‘Efficiencies’ file. **(d)** URLs are generated (‘URL’ file) which allow visualization of the reads in the freeware UCSC Genome Browser database^26^. However, if the number of total reads (also the reads that are discarded by the tool) exceeds 1000, visualization via UCSC is no longer possible. As an alternative, raw NGS result files (fastQ) can be uploaded into the Integrative Genomics Viewer (IGV)^19^,^20^.

For each entry in the experiment file, a folder with four output files is generated, containing genome editing data for every sample analysed for the user-defined region of interest (Fig. 2). The ‘Variants’ text file lists the chromosome, chromosomal location, type (insertion or deletion), length, the flanking sequence of the indel and absolute and relative frequency of each variant, initiated in the region of interest. In case HDR analysis is requested, a ‘RepairReport’ file is generated, providing information on the type and frequency of HDR events. In addition, general indel and repair rates are calculated and shown in the ‘Efficiencies’ file. Finally, URLs are generated (‘URL’ file) which allow visualization of the reads in the UCSC Genome Browser^22^.

### Validation performance of BATCH-GE

To display the potential of BATCH-GE, two genome editing experiments were conducted in zebrafish (see details in Supplementary results). First, we aimed to determine optimal experimental conditions for sgRNA efficiency testing in zebrafish embryos. For this purpose, sgRNAs targeting five different zebrafish genes (*slc2a10*, *pls3*, *tapt1a*, *myt1la*, *tprkb*), were injected into one-cell stage zebrafish embryos, to both determine the most optimal ratio of sgRNA to Cas9 and the most relevant developmental time point for indel efficiency analysis. Genome editing assessment using the BATCH-GE tool revealed that combinations of 10 pg sgRNA + 250 pg Cas9 and 25 pg sgRNA + 250 pg Cas9 resulted in the highest indel frequencies, suggesting that Cas9 is the determining factor when aiming to achieve high indel rates (Supplementary Fig. S2). These results correspond to earlier findings in other organisms, showing a positive correlation between Cas9 quantities and indel efficiency^23^,^24^,^25^,^26^. Furthermore, the data shows that genome editing analysis of DNA extracted at 1 dpf results in a reliable estimation of the indel efficiency at later stages during zebrafish development^27^,^28^.

In a second experiment, we aimed to introduce specific base pair alterations in the zebrafish *tprkb* gene. First, we validated and further optimized a protocol by Irion *et al.*^29^, describing a strategy for the achievement of CRISPR/Cas9-mediated precise genome editing by HDR in zebrafish, using a circular HDR template. In a second approach, short linear single-stranded oligodeoxynucleotides (ssODN) were screened for their suitability as HDR template^30^,^31^. Four ssODN, either sense or antisense relative to the sgRNA sequence identity and with 30 or 60 bp homology arms, that are flanking the theoretical CRISPR/Cas9 cut site, were designed (Supplementary Fig. S3). For precise genome editing analysis, BATCH-GE requires the specification of a repair template in the Experiment.csv file (Supplementary Fig. S1). Brackets were placed around the 5 or 6 intended base pair alterations. By placing square or round brackets around these base pair substitutions, BATCH-GE is able to distinguish between the occurrence of a ‘full’ or a ‘partial’ repair. Square brackets indicate the base pair alterations that need to be introduced in the zebrafish genome while round brackets indicate alterations that do not necessarily need to be introduced in the genome, for instance base pair alterations that are used for codon optimization of the template. Reads that only contain the necessary alterations and reads that contain all the indicated base pair alterations are classified and counted as partial and full HDR events respectively. In general, two conclusions can be drawn from the BATCH-GE output (Supplementary Table S1). First, as already shown by Irion *et al.*^29^, HDR efficiencies are relatively low when using the described circular templates. Secondly, the use of ssODN repair templates leads to improved total repair efficiencies, similar to those described earlier^30^,^31^, especially when using templates with 60 bp homology arms.

### Influence of the user-defined region-of-interest size on indel efficiency calculation

**Figure 3:**
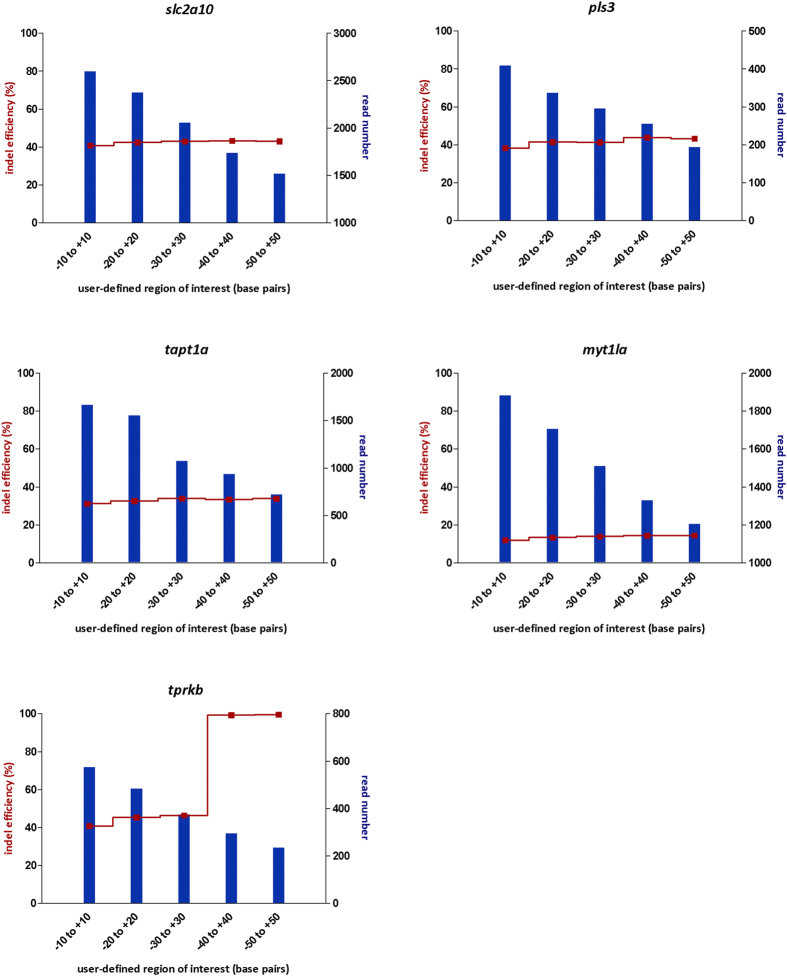
Indel rates and read number, as a function of the size of the region of interest used in BATCH-GE. The raw sequencing data derived from CRISPR/Cas9 assays (*slc2a10*, *pls3*, *tapt1a*, *myt1la*, *tprkb*) injected with 25 pg sgRNA and 250 pg Cas9 and analysed at 1 dpf were reanalysed while varying the size of the region of interest from 20 to 100 bp. The blue bars represent the number of reads retained by BATCH-GE when screened for coverage of the user-defined region of interest. The red line represents the indel rate as a function of the size of the region of interest.

BATCH-GE requires per amplicon a user-defined region of interest comprising the theoretical CRISPR/Cas9 cut site, which is specified in the ‘Cutsites.bed’ file (Table 2). It is anticipated that the size of this region, as defined in the BED file, influences the number of reads included in the analysis. The larger the genomic region (region of interest) the reads need to cover, the lower the number of reads that will be retained in the analysis by BATCH-GE. To find out if a change in read number influences the indel rate calculation, the dimension of the region of interest was varied from 20 base pairs up to 100 base pairs (position −10 to +10 and −50 to +50 respectively, considering the theoretical CRISPR/Cas9 cut site as reference point) when analysing the sequencing data from the CRISPR/Cas9 assays that were previously used to determine optimal experimental conditions for sgRNA efficiency testing in zebrafish (Supplementary Fig. S2). As expected, the number of reads included in the analysis dropped significantly during the transition from a 20 bp to a 100 bp region of interest (Fig. 3). Therefore, intuitively, one would opt for selecting a 20 bp (−10 to +10) region of interest, in this way maximizing the number of reads included in the analysis. Previously, Varshney *et al.*^32^ reported that the majority of indel mutations introduced in the zebrafish germline does not exceed a size of 20 base pairs, although CRISPR/Cas9 can also introduce indel mutations far bigger than 20 base pairs. In accordance, a minor increase in indel efficiency could be noticed in the majority of the assays, when increasing the size of the region of interest from 20 bp to 100 bp (Fig. 3). Therefore, selecting a 20 bp region of interest would possibly result in a slightly underestimated indel rate. Selecting a large region of interest on the other hand, might result in a fairly low number of reads included in the analysis, which could influence the reliability of the generated results. Altogether, considering these results, when using BATCH-GE, selecting a dimension between the two extremes, for instance 60 bp (−30 to +30) is suitable for most of the experiments.

Remarkably, an abrupt increase in indel efficiency could be observed for the *tprkb* assay when selecting an 80 to 100 bp region of interest (Fig. 3). In this particular case, the region of interest (ROI) was expanded into intronic sequences, where the studied zebrafish population harboured a highly frequent 1 bp insertion polymorphism, explaining the nearly 100% indel rates for these regions of interest. Therefore, it is advisable to either incorporate a control sample (e.g. non-injected zebrafish embryos from the same clutch) in the analysis to identify these polymorphisms, or to examine the reads visually using the UCSC links in the URL.txt file to control for the possible incorporation of intronic regions and inherent indel polymorphisms in the screened region of interest.

## Discussion

CRISPR/Cas9 genome editing is currently revolutionizing genetics and has enabled labs from all over the world to conduct nuclease-mediated genome engineering experiments. In addition, the simplicity of this technique has enabled the high-throughput set-up of CRISPR-Cas9-based experiments. Thus, analysing these experiments is a challenging and time-consuming task. NGS has been replacing standard techniques such as the Surveyor’s, the T7 Endonuclease I assay and the sequencing of cloned PCR products, due to its high capacity, sensitivity, the increasing accessibility and the ever-decreasing cost. According to De Leeneer *et al.*^33^, NGS sequencing costs dropped to less than one tenth of Sanger sequencing costs. However, the analysis of large amounts of NGS data is a significant hiatus in the otherwise advanced field of CRISPR/Cas9. In this work, we present and evaluate a straightforward tool, BATCH-GE, for large-scale analysis of genome editing experiments. BATCH-GE shows important advantages over the current NGS-based genome-editing analysis methods. The tool’s most striking asset and improvement over the existing tools, such as CRISPR-GA, is that it allows for batchwise analysis of a large number of samples, which is time-efficient in terms of effective hands-on time (Table 3). Moreover, BATCH-GE is implemented as a freely available Perl script, and is therefore available for further optimization by the user. In addition, it can run either within a server environment or on a stand-alone computer, providing an ensured availability. Furthermore, the user is only required to complete two simple input files (a comma separated experiment file and a BED file containing the user-defined regions of interest) prior to the analysis, containing easy-to-determine variables such as sample identifier, genomic coordinates and directory paths. Additionally, the tool provides the user with the necessary flexibility, since the input files contain a number of user-defined variables, such as the size of the genomic region of interest surrounding the theoretical CRISPR/Cas9 cut site and the sequence composition of the repair template. First, the size of the region of interest influences the number of reads included in the analysis and consequently the calculated indel rate. The number of reads can be highly variable between different experiments and decreasing the region of interest size will increase the number of retained reads. In general, the indel rate shows little variation when changing the size of the region of interest and consequently the number of retained reads. A second user-defined variable is the sequence composition of the repair template. Specific base pair alterations in the repair template, which is a required input variable for HDR analysis, can be marked, allowing the simultaneous detection of single and multiple intended base pair substitutions. A distinction can be made between base pair changes that are absolutely required to be introduced in the genome and alterations that should not necessarily be included. The latter type of alteration could, for instance, be needed to codon-optimize the sgRNA target sequence in the repair template. In other words, BATCH-GE can distinguish between a full and a partial repair in one single analysis, in this way providing distinctive information about necessary and unnecessary genomic alterations.

BATCH-GE generates four comprehensive text files. First, the Variants.txt file lists genomic region, type, length, the flanking sequence of the indel and frequency of every detected indel variant per sample, in this way providing a detailed overview of all sequence alterations. Second, HDR efficiency is evaluated in the RepairReport.txt file, providing a distinctive analysis of full and partial HDR events. Third, the Effiencies.txt file summarizes general indel and repair rates, enabling a quick and straightforward evaluation of the overall mutation efficiency. Fourth, a URL.txt file is generated, providing URLs to visualize the reads in the UCSC genome browser, offering the possibility to look into the specific sequence alterations.

Finally, to evaluate the performance of BATCH-GE, the tool was used for genome editing assessment in two zebrafish experiments, covering sgRNA efficiency testing and precise genome editing via HDR. Previously, an influence of sgRNA and Cas9 quantities on indel efficiencies was reported in several organisms^23^,^24^,^25^,^26^. Using BATCH-GE, this finding was confirmed when comparing general indel rates after injection of four combinations of sgRNA (RNA) and Cas9 (protein) quantities in zebrafish; higher Cas9 quantities consequently led to higher indel rates. In addition, it was noted that indel rates remained relatively stable from 1 dpf on, a finding that was also reported earlier^27^,^28^. In a second validation experiment, the introduction of specific base pair alterations in zebrafish via HDR was intended^29^,^34^. For both circular and ssODN HDR templates, BATCH-GE generates similar repair efficiencies as already reported in literature^29^,^30^,^31^.

In conclusion, BATCH-GE is a new tool for the analysis of NGS-derived genome editing data and contributes to a faster, more informative and flexible analysis of multiple genome editing experiments for virtually any organism of interest.

## Methods

### sgRNA design and production

For 5 different zebrafish genes (*pls3*, *slc2a10*, *tapt1a*, *myt1la*, *tprkb*), single-guide RNA (sgRNA) sequences were designed using the CRISPRdirect software (http://crispr.dbcls.jp/)^35^. Refseq mRNA accession numbers or mRNA sequences were used as input and the ‘NGG’ PAM sequence was selected. For each gene, a sgRNA target sequence was selected, guided by a number of criteria, which are based on current knowledge on optimal sgRNA design and which are listed in Supplementary Table S2.

For the selected sgRNA target sequences, a synthetic double-stranded DNA molecule (gBlock, IDT) was constructed, with the following sequence: 5′-CCGCTAGCTAATACGACTCACTATA-**GG**-**N**_**18**_-GTTTTAGAGCTAGAAATAGCAAGTTAAAATAAGGCTAGTCCGTTATCAACTTGAAAAAGTGGCACCGAGTCGGTGCTTTT-3′ where N_18_ represents the protospacer sequence. A schematic overview of the gBlock design is depicted in Supplementary Fig. S3 and the gBlock sequences are listed in Supplementary Table S3. Two hundred ng gBlock DNA molecules were dissolved in 20 μl nuclease-free water. *In vitro* transcription was carried out using the MEGAshortscript™ T7 Transcription Kit (Invitrogen, AM1354), following the general guidelines of the manufacturer. An input volume of 4 μl dissolved DNA (10 ng/μl) was used and an overnight incubation step at 37 °C was performed to obtain a maximum yield. To purify the transcription reaction, the MEGAclear™ Kit (Life Technologies, AM1908) was used, following the manufacturer’s instructions. The resulting RNA was quantified with a DropSense96 device (Trinean), checked for integrity using the Experion microfluidic capillary electrophoresis system (Bio-Rad), aliquoted and stored at −80 °C.

### Homology-directed repair template design and production

Plasmid HDR templates were designed as synthetic double-stranded DNA molecules (gBlock, IDT). The sequence and a schematic overview of the synthetic DNA molecule are displayed in Supplementary Fig. S3. The synthetic DNA molecules were PCR amplified with primers 5′GTGGACTGGATTGCCATTCT3′ and 5′GCGAAGCCACACTTCCAA3′ using the KAPA2G Robust HotStart ReadyMix (KK5702, Kapa Biosystems) and cloned into the pGEM®-T Easy Vector (A1360, Promega). The insert sequence was confirmed with Sanger sequencing using the following primers: 5′GGATCTGGAACGCATCTACAA3′, 5′TGACGTTTCTGAAAACAAGACAA3′, 5′TCTTTGAACTGTGGGGGAAA3′, 5′GGGTCAGATGGCATTTCTGT3′, 5′CACACTTCCAAGTCCACCAG3′ and 5′AGAACATGTAAAGCACTGTTTC3′. Single-stranded oligodeoxynucleotides (ssODN) were designed and ordered as ultramer oligonucleotides, without PAGE purification (4 nmol, IDT). The sequence and a schematic overview of the ssODN molecules are displayed in Supplementary Fig. S3.

### Zebrafish handling and embryo injection

Zebrafish handling, mating, embryo collection and maintenance was conducted as described earlier^36^, in agreement with EU Directive 2010/63/EU for animals. Approval for this study was provided by the local committee on the Ethics of Animal Experiments (Ghent University Hospital, Ghent, Belgium; Permit Number: ECD 14/31). All methods were carried out in accordance with the approved guidelines. One-cell stage zebrafish embryos were microinjected in the cell with 1,4 nl mix containing 10 or 25 pg sgRNA, 100 or 250 pg Cas9 protein (Cas9 wild type nuclease protein with NLS, ToolGen) and, in specific experiments, a plasmid (100 pg) or ssODN (50 or 100 pg) HDR template, complemented with RNase-free water and phenol red sodium salt indicator (P4758, Sigma Aldrich).

### Genomic DNA extraction

For every experiment, genomic DNA extraction was carried out on a pool of 15 injected, dechorionated embryos, using KAPA Express Extract DNA Extraction Kit (Kapa Biosystems, KK7103). Extraction mixes consisting of 15 embryos, 10 μl 10X Kapa Express Extract Buffer, 2 μl Express Extract Enzyme (1 U/μl) and 88 μl PCR grade water were incubated at 60 °C for 10 minutes and 95 °C for 5 minutes. The supernatant resulting from 1 minute full speed centrifugation was collected and stored at −20 °C.

### Next generation sequencing and data analysis

Extracted DNA was singleplex PCR-amplified using target-specific primers (Supplementary Table S4). PCR products were integrated in the library preparation and sequenced on an Illumina MiSeq platform according to De Leeneer *et al.*^33^. Following NGS, BATCH-GE directly used raw NGS data to generate a detailed report on mutagenesis efficiencies. Raw reads were trimmed with Q30 cut-off using the FASTX-Toolkit and mapped to the reference genome using the BWA MEM algorithm. After duplicate read removal (Picard Tools), a Sequence Alignment Map (SAM) file was generated. From this file, the BATCH-GE algorithm screened the reads for the presence of mutations or specific base pair alterations. The rationale behind this analysis is visualized in Fig. 1. The algorithm behind BATCH-GE is implemented in a Perl script (Perl 5). We created a standalone package including the script, all dependencies for installation and two example datasets, which is freely available for academic use and can be downloaded from https://github.com/WouterSteyaert/BATCH-GE.git using the installation notes that can be found in the Supplementary information as well as in the README file. BATCH-GE is developed for Linux OS and is successfully tested on Ubuntu 12, Ubuntu 14 and Debian 8 Linux distributions. To run the software on Windows or Mac, we advise to use a virtualization software such as Virtual Box.

## Additional Information

**How to cite this article**: Boel, A. *et al.* BATCH-GE: Batch analysis of Next-Generation Sequencing data for genome editing assessment. *Sci. Rep.* **6**, 30330; doi: 10.1038/srep30330 (2016).

## Supplementary Material

Supplementary Information

## Figures and Tables

**Table 1:** BATCH-GE input files: the Experiment.csv file.

FastqDir	Sample Numbers	Genome	CutSite	OutputDir	CutSites File	Repair Sequence
/location of the FastQ files/	a, b, f, g, h	Genome A	Gene A amplicon 1	/location of the output files/runX/	/location of the CutSites file/Cutsites.bed	(N)NNNNNN(N)NNNNNNNNNN(N)NNNNNNNNNNNN(N)NNNN[N]
/location of the FastQ files/	a–z	Genome A	Gene A amplicon 2	/location of the output files/runY/	/location of the CutSites file/Cutsites.bed	/
/location of the FastQ files/	a, b, d–g	Genome B	Gene B	/location of the output files/runZ/	/location of the CutSites file/Cutsites.bed	/

The experiment file contains all information that is specific for the experiment. The mandatory headers are 1) FastqDir, i.e. the full path of the directory of the FastQ files, 2) SampleNumbers, i.e. identifier(s) for the sample(s) to be analysed in the specified NGS sequencing run. The notation x,y ensures that both samples x and y will be analysed. If numerical identifiers are used the notation x-y means that all samples from x to y will be analysed, 3) Genome, i.e. the build of the reference genome the reads should be mapped to (cf. installation notes), 4) CutSite, i.e. identifier of choice for the particular cut site. This identifier needs to be the same as the identifier mentioned in the BED file (4^th^ column), 5) OutputDir, i.e. the directory where the output of BATCH-GE should be stored and 6) Location CutSites File, i.e. BED file containing the genomic coordinates of all cut sites used in the experiment. An optional header is RepairSequence, in this column the HDR template sequence must be placed. Placing square brackets around certain bases of the repair template indicates that these base pair alterations need to be introduced in the zebrafish genome. Round brackets on the other hand, indicate base pair alterations that do not necessarily need to be introduced in the genome e.g. alterations needed for codon optimization of the template.

**Table 2:** BATCH-GE input files: the Cutsites.bed file.

Chromosome	Chromosomal position start region of interest	Chromosomal position end region of interest	Designation region of interest
chrA	Theoretical cut site −30	Theoretical cut site +30	Gene A amplicon 1
chrA	Theoretical cut site −30	Theoretical cut site +30	Gene A amplicon 2
chrB	Theoretical cut site −30	Theoretical cut site +30	Gene B

The designation of the user-defined region of interest indicated in the ‘CutSite’ column of the Experiment.csv file, can be specified through the ‘cutsite.bed’ file. Each row represents one region of interest and contains the chromosome, user-defined chromosomal start and end position and the designation of the region of interest (should be identical to the names in the ‘CutSite’ column of the ‘Experiment.csv’ file). No header should be included. In general, in this file, the user can specify the region of interest surrounding the theoretical CRISPR/Cas9 cut site. This is generally a region of 20 (position −10 to +10, relative to the theoretical CRISPR/Cas9 cut site) to 100 (−50 to +50) base pairs.

**Table 3:** Comparison between available tools for analysis of NGS-derived genome editing data.

	Visualization in genome browser	CRISPR-GA	BATCH-GE
Freely available	*x*	*x*	*x*
Mapping reads against complete genomic sequence of the organism of interest	*x*		*x*
Specific analysis genome editing experiment		*x*	*x*
Calculation rate mutagenic events		*x*	*x*
Graphical interpretation mutagenic events		*x*	
Generation list of specific variants			*x*
Distinction between full and partial HDR			*x*
Adjustment input parameters			*x*
Analysis multiple samples in batch			*x*
