# Publisher Correction: BATCH-GE: Batch analysis of Next-Generation Sequencing data for genome editing assessment

**DOI:** 10.1038/s41598-018-33869-y

**Published:** 2018-10-29

**Authors:** Annekatrien Boel, Wouter Steyaert, Nina De Rocker, Björn Menten, Bert Callewaert, Anne De Paepe, Paul Coucke, Andy Willaert

**Affiliations:** Center for Medical Genetics, Ghent University Hospital, Ghent, Belgium

Correction to: *Scientific Reports* 10.1038/srep30330, published online 27 July 2016

This article contains a typographical error in the spelling of the author Wouter Steyaert, which is incorrectly given as Woutert Steyaert.

