# Supplementary material for: BATCH-GE: Batch analysis of Next-Generation Sequencing data for genome editing assessment
Source: Sci Rep. 2016 Jul 27;6:30330. doi: 10.1038/srep30330 (PMC4962088; doi:10.1038/srep30330)
Supplement: Supplementary Information [file srep30330-s1.pdf]

SUPPLEMENTARY INFORMATION

BATCH-GE: Batch analysis of Next-Generation Sequencing data for genome editing assessment

Boel Annekatrien [1,¶] , Steyaert Wouter [1 ,¶], De Rocker Nina [1], Menten Björn [1], Callewaert Bert [1], De Paepe Anne [1], Coucke Paul [1], Willaert Andy [1,*]

[1] Center for Medical Genetics, Ghent University Hospital, Ghent, Belgium

[¶] These authors contributed equally to this work.

* To whom correspondence should be addressed (Andy.Willaert@UGent.be).

Supplementary results

Validation performance of BATCH-GE

To display the potential of BATCH-GE, two genome editing experiments were conducted in zebrafish. First, we aimed to determine optimal experimental conditions for sgRNA efficiency testing in zebrafish embryos. For this purpose, sgRNAs targeting five different zebrafish genes (*slc2a10*, *pls3*, *tapt1a*, *myt1la*, *tprkb*), were designed and produced according to an in-house developed workflow using synthetic dsDNA templates (Supplementary Fig. S3). For these assays, both the most optimal ratio of sgRNA to Cas9 and the most relevant developmental time point for indel efficiency analysis were determined. This was achieved by injecting 4 different combinations of sgRNA (10 and 25pg) and Cas9 (100 and 250pg) quantities in one-cell stage zebrafish embryos, followed by DNA extraction of a pool of injected embryos at 1, 2, 3 and 4 days post fertilization (dpf). Genome editing assessment using the BATCH-GE tool revealed that combinations of 10 pg sgRNA + 250 pg Cas9 and 25 pg sgRNA + 250 pg Cas9 resulted in the highest indel frequencies, suggesting that Cas9 is the determining factor when aiming to achieve high indel rates (Supplementary Fig. S2). These results correspond to earlier findings in other organisms, showing a positive correlation between Cas9 quantities and indel efficiency [1-4]. Furthermore, the data shows that genome editing analysis of DNA extracted at 1 dpf results in a reliable estimation of the indel efficiency at later stages during zebrafish development [5,6].

In a second experiment, we aimed to introduce specific base pair alterations in the zebrafish tprkb gene. First, we validated and further optimized a protocol by Irion et al. (2014) [7], describing a strategy for the achievement of CRISPR/Cas9-mediated precise genome editing by HDR in zebrafish. This strategy involves the use of a circular HDR template which was produced according to an in-house developed workflow using synthetic dsDNA templates (Supplementary Fig. S3), and was co-injected with Cas9 nuclease and an sgRNA that was previously shown to be highly efficient in targeting the zebrafish tprkb gene (Supplementary table S3, design 2). After injection in the embryo, the circular

template is cut at two sgRNA target sequences (+PAM) which are flanking a sequence, homologous to the genomic target site, hence providing a linearized HDR template [7]. In a second approach, short linear single-stranded oligodeoxynucleotides (ssODN) were screened for their suitability as HDR template[8,9]. Four ssODN, either sense or antisense relative to the sgRNA sequence identity and with 30 or 60 bp homology arms, that are flanking the theoretical CRISPR/Cas9 cut site, were designed (Supplementary Fig. S3). For both types of HDR templates, the highest amount that did not cause any toxic effects (plasmid: 100 pg, ssODN: 50-100 pg) was injected in one-cell stage zebrafish embryos together with 25 pg sgRNA and 250 pg Cas9. DNA was extracted at 1 dpf and analysed using NGS, followed by genome editing assessment using BATCH-GE. For precise genome editing analysis, BATCH-GE requires the specification of a repair template in the Experiment.csv file (Supplementary Fig. S1). Brackets were placed around the 5 or 6 intended base pair alterations. By placing square or round brackets around these base pair substitutions, BATCH-GE is able to distinguish between the occurrence of a 'full' or a 'partial' repair. Square brackets indicate the base pair alterations that need to be introduced in the zebrafish genome while round brackets indicate alterations that do not necessarily need to be introduced in the genome, for instance base pair alterations that are used for codon optimization of the template. Reads that only contain the necessary alterations and reads that contain all the indicated base pair alterations are classified and counted as partial and full HDR events respectively. In general, three conclusions can be drawn from the BATCH-GE output (Supplementary Table S1). First, as already shown by Irion et al. (2014) [7], HDR efficiencies are relatively low when using the described circular templates. Secondly, the use of ssODN repair templates leads to improved total repair efficiencies, similar to those described earlier [8,9], especially when using templates with 60 bp homology arms. Thirdly, no difference in HDR efficiency could be detected between sense and antisense ssODN HDR template molecules.

Figures

Supplementary Figure 1: Printscreens of specified BATCH-GE input files for the experiments conducted to validate the performance of BATCH-GE (a) The majority of experimental specifications are supplied to BATCH-GE via the Experiment.csv file. Beside a header, each line of the file, represents one NGS run and contains the following information: 1) the full path of the directory of the FastQ files (FastqDir), 2) Numbers of pools that are analyzed for the same region of interest (SampleNumbers), 3) genome release of the organism of interest (Genome), 4) designation of the user-defined region of interest (CutSite), which serves as the first link between the 'Experiment.csv' file and the 'Cutsites.bed' file, 5) the desired location and name of the output folder, which will be generated automatically (OutputDir), 6) the location of the 'Cutsites.bed' file, which serves as a second link between the Experiment.csv and the Cutsites.bed file (CutSitesFile), 7) the repair template sequence in case of the need to screen for HDR events (RepairSequence). **(b)** The designation of the user-defined region of interest indicated in the CutSite column of the Experiment.csv file, can be specified through the Cutsites.bed file. Each line represents one region of interest and contains the chromosome, user-defined chromosomal start and end position and the designation of the region of interest (identical to the names in the CutSite column of the Experiment.csv file), separated by a tab. No header should be included. In other words, in this file, the user can specify the region of interest surrounding the theoretical CRISPR/Cas9 cut site. This is generally a region of 20 (position -10 to +10, relative to the theoretical CRISPR/Cas9 cut site) to 100 (-50 to +50) base pairs. The Cutsites.bed file can be used as a library for all sgRNA cut sites for one organism. When this file contains all sgRNA cut sites for which one generally analyzes samples, this file should never be changed before running a particular analysis.

a

```
FastqDir;SampleNumbers;Genome;CutSite;OutputDir;CutSitesFile;RepairSequence
/home/BATCH-GE/Fastq/run94/;1-16;danRer7;slc2a10;/home/BATCH-GE/run94/slc2a10/;/home/BATCH-GE/ZebrafishCutsites.bed
/home/BATCH-GE/Fastq/run94/;1-16;danRer7;pls3;/home/BATCH-GE/run94/pls3/;/home/BATCH-GE/ZebrafishCutsites.bed
/home/BATCH-GE/Fastq/run94/;1-16;danRer7;tapt1a;/home/BATCH-GE/run94/tapt1a/;/home/BATCH-GE/ZebrafishCutsites.bed
/home/BATCH-GE/Fastq/run94/;1-16;danRer7;myt1la;/home/BATCH-GE/run94/myt1la/;/home/BATCH-GE/ZebrafishCutsites.bed
/home/BATCH-GE/Fastq/run94/;1-16;danRer7;tprkb.1;/home/BATCH-GE/run94/tprkb.1/;/home/BATCH-GE/ZebrafishCutsites.bed
/home/BATCH-GE/Fastq/run102/;9,12,15;danRer7;tprkb.2;/home/BATCH-GE/run102/tprkb.2/;/home/BATCH-GE/ZebrafishCutsites.bed;(A)GATGC(G)TTCCAGATCCT(T)GT(C)GCAACAAATAAAGCAGTTCA[G]
/home/BATCH-GE/Fastq/run115/;11-14;danRer7;tprkb.2;/home/BATCH-GE/run115/tprkb.2/;/home/BATCH-GE/ZebrafishCutsites.bed;(C)GCATT(T)CAGATCCT(C)GT(A)GC(C)AC[C]
```

b

```
chr11	2431364 2431423 slc2a10
chr14	13724028	13724087	pls3
chr14	49587123	49587182	tapt1a
chr20	30559186	30559245	myt1la
chr4	27683449	27683508	tprkb.1
chr4	27683446	27683505	tprkb.2
```

Supplementary Figure 2: Determination of the most optimal experimental conditions for indel efficiency analysis in zebrafish CRISPR/Cas genome editing experiments. For a subset of five randomly selected designs (*slc2a10*, *pls3*, *tapt1a*, *myt1la*, *tprkb*), indel efficiency (%) was determined at different time points for different injection quantities of sgRNA and Cas9. Low (blue) and high (red) sgRNA concentrations were mixed with low (dashed line) and high (full line) Cas9 concentrations.

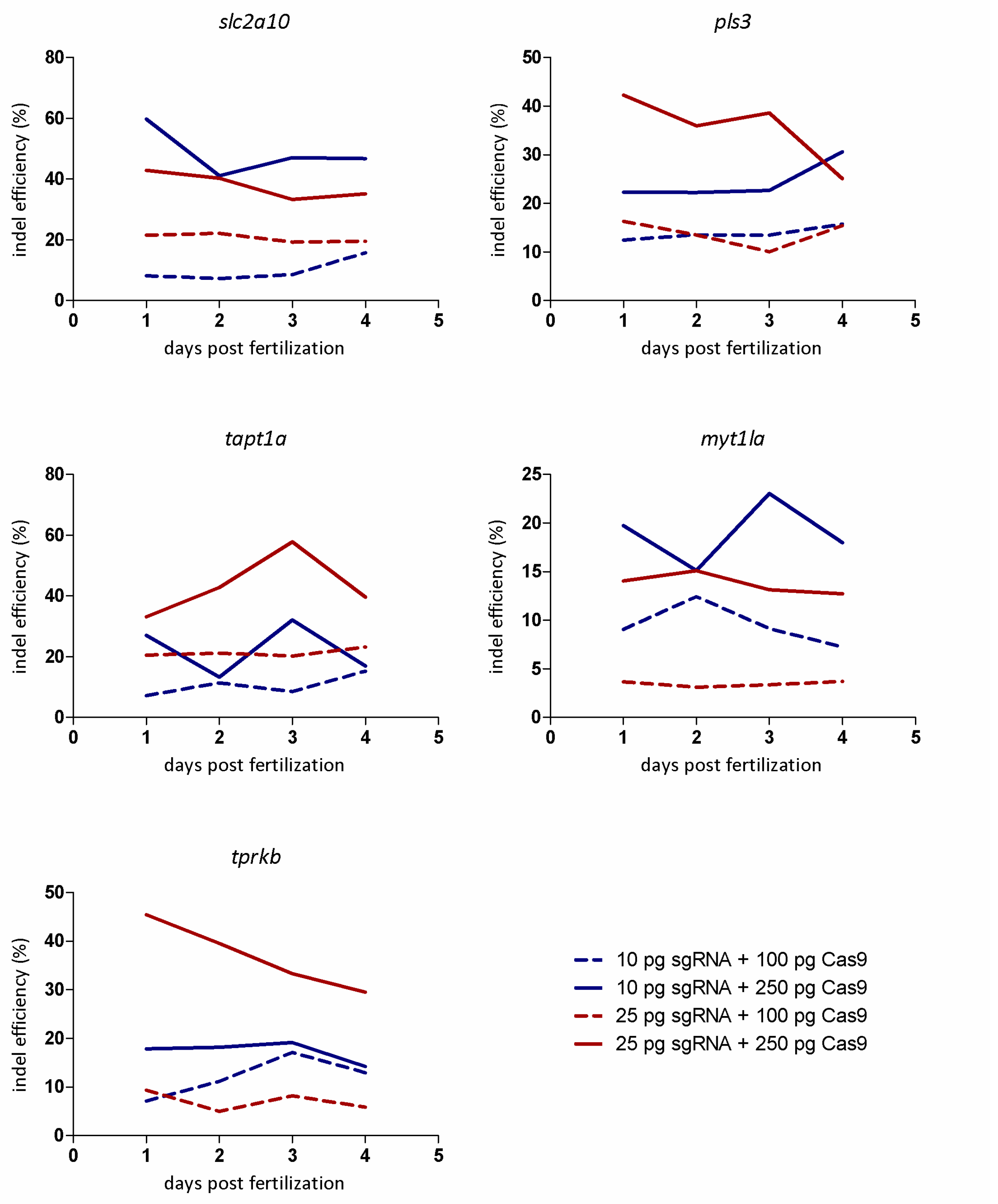

Supplementary Figure 3: Schematic overview and nucleotide sequence of the synthetic double-stranded DNA used to generate sgRNAs and HDR templates that target the zebrafish *tprkb* gene (a) Schematic representation and nucleotide sequence of the sgRNA template. Synthetic double-stranded (ds) DNA sequences consist of a random sequence to facilitate the initiation of *in vitro* transcription (IVT), a T7 promoter necessary for IVT with T7 RNA polymerase, and a sgRNA encoding sequence containing 3 parts: two guanines, base pairs 3 to 20 of the selected protospacer (displayed here as 'N') and an invariable sequence. **(b)** Schematic representation and nucleotide sequence of the HDR template according to Irion *et al.* (2014) [7]. The synthetic DNA molecules consist of a 900 bp sequence, homologous to the zebrafish *tprkb* gene, flanked by sgRNA target sequences (+ PAM) to obtain linearized DNA molecules *in vivo*, and 50 bp random DNA sequences, to facilitate gBlock construction. The 900 bp homologous sequence contains 5 base pair substitutions (designated in bold). Central in the homologous sequence is the sgRNA target site located. To avoid unintended HDR template cleavage in the 900 bp homologous sequence, the sgRNA target sequence (+ PAM) is codon optimized by altering 4 base pairs (base pairs in bold and underlined). In addition, an intended base pair substitution is inserted 25 base pairs downstream from the theoretical cut site (c.189C>G) (in bold and underlined). The displayed HDR template was co-injected with an sgRNA with protospacer sequence 5' GGATGCATTCCAGATCCTGG 3' **(c)** Schematic representation and nucleotide sequence of 4 single-stranded oligodeoxynucleotides (ssODN) HDR template molecules, sense or the antisense relative to the sgRNA sequence identity and with either 30 or 60 bp homology arms flanking the theoretical CRISPR/Cas9 cut site. The 6 base pair substitutions are designated in bold. The displayed HDR templates were co-injected with an sgRNA with protospacer sequence 5' GGATGCATTCCAGATCCTGG 3'.

a

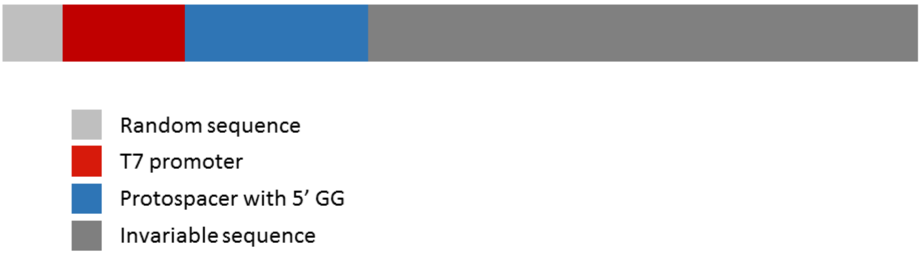

CCGCTAGCTAATACGACTCACTATAGGGCGTCAATAATCGGCGGCGTTTTAGAGCTAGAAATAGCAAGTTAAAATAAGGCTAGTCCGTTATC
AACTTGAAAAAGTGGCACCGAGTCGGTGCTTTT

b

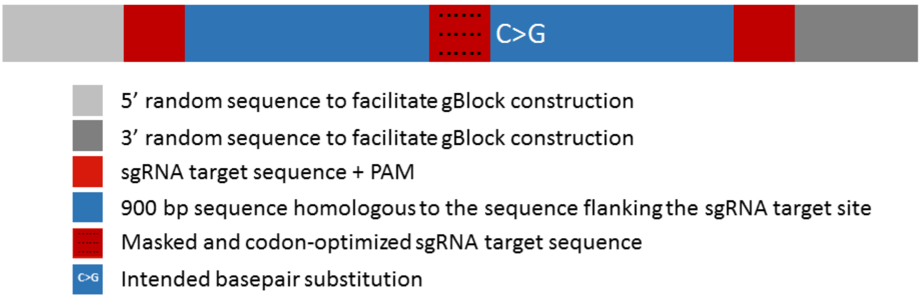

CGGGTCACGGCTTGTCGTGGACTGGATTGCCATTCTCTGAGTGTATTACGGGATGCATTCCAGATCCTGGTGGACATAAACAACACAGACA
TGGTCAGATAATACTCTTAAGTTAATGCATATTGCAGAACATAATCAATTCTCTCAAAGAATCTCACCGTGTATCTCCTCACTTGCAAAATATTT
GTTAGAGTAAAATATTTGTTTTATTCTATGAATGTTTAAACCTATTTCAGTGAACATAATTTAAAGCTTCTGTTTATTTGTTGAAGAAAATAACA
TTTTGGCATAATACTGAAATGTTTTCATTTGTTATTGTTGAAATCAGTGTTATTCCACCAAATATTGATTGCTTACATCTTCTGAAGTAAAAAAC
ATTTAAACATTCAGTATTGTGTTGTCTAAAATTATATGACGTTTCTGAAAACAAGACAAATAATAGTCAAAATATATTCTAAAAACATGTACGTA
TATTTACCTTTTTATTTTTTTTTAATAAAGGTTGTAGATGCGTTCCAGATCCTTGTCGCAACAAATAAAGCAGTTCAGCTTCATAAAATCGGGA
AAATGAAAACCAGAAGCCTTTACTCTGAAATCATTTTCAATCTTTCACCAACAAACAATGTAAGTAACATTTCAAATACCTTAGTGGCTCAGT
GGTTAGCACTGTCATCTCACAGCAAGAAGGTCACTGGTTCAAGTCCCGGCTGGGTCAGATGGCATTTCTGTGTGGAGTTTGCATGTTCTCC
CTATGTTGGCGTGGGTTTCCTCCAGTTTCCCCCACAGTTCAAAGACATGTGGTATAGGTGAATTGAATAAGCTAAATTGGTCGTAGTGTATG
GGTGTGAATGAGAGTGTATGGATGTTTACCAGTACTGGGTTGCAGCTTGAAGGGCATCTGCTGTGTAAAACATATGCTGGATAAGTTGGTG
GTTCATTCCTCTGTGGTGACCCCTGATAACTAAAGGAACTAGGATGCATTCCAGATCCTGGTGGACTTGGAAGTGTGGCTTCGCCTTTGCC
CACGCGCCTGGTCGCTCCTCGTT

c

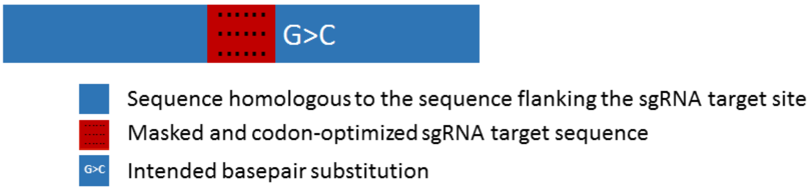

sense, 30 bp homology arms

TAATAAAGGTTGTGGACGCATTTCAGATCCTCGTAGCCACCAATAAAGCAGTTCACCTTC

sense, 60 bp homology arms

TGTACGTATATTTACCTTTTTATTTTTTTTTAATAAAGGTTGTGGACGCATTTCAGATCCTCGTAGCCACCAATAAAGCAGTTCACCTTCATAA
AATCGGGAAAATGAAAACCAGAAGCC

antisense, 30 bp homology arms

GAAGGTGAACTGCTTTATTGGTGGCTACGAGGATCTGAAATGCGTCCACAACCTTTATTA

antisense, 60 bp homology arms

GGCTTCTGGTTTTCATTTTCCCGATTTTATGAAGGTGAACTGCTTTATTGGTGGCTACGAGGATCTGAAATGCGTCCACAACCTTTATTAAA
AAAAAATAAAAAGGTAAATATACGTACA

Tables

Supplementary Table 1: Precise genome editing in the zebrafish *tprkb* gene. Maximum quantities of HDR repair templates, homologous to the *tprkb* gene, with no detectable toxic effect on development, were co-injected with an sgRNA targeting the *tprkb* gene (design #2) and Cas9, after which the corresponding indel and repair efficiency were determined with BATCH-GE.

Nature HDR template	Injection quantity (pg)	Indel efficiency (%)	Total Repair efficiency (%)	Full repair (number of reads)	Partial repair (number of reads)	Total number of reads
**plasmid, 450 bp homology arms**	100	86,00	0,78	1	4	643
**ssODN, sense, 30 bp homology arms**	50	90,78	0,56	7	0	1247
**ssODN, sense, 60 bp homology arms**	100	74,59	5,61	17	0	303
**ssODN, antisense, 30 bp homology arms**	50	77,12	0,37	0	1	271
**ssODN, antisense, 60 bp homology arms**	100	74,15	5,44	7	1	147

Supplementary Table 2: Selection criteria for sgRNA design. Criteria reported to be useful in avoiding off-target effects and increasing indel mutation rates. As the CRISPR/Cas technology continues to evolve, the list of criteria may be extended and adapted in the future.

sgRNA selection criteria	Motivation	Reference
When using CRISPRdirect software: select designs with number of target sites for complete target sequence + PAM=1 and for 12 base pair seed sequence+PAM=1.	Designs with unique 20-mer and 12-mer seed sequence, will aid in avoiding off-target effects.	[1,2,10-14]
Select designs at the 5' end of the gene.	The aim is to terminate translation as early as possible and to induce the nonsense-mediated mRNA decay pathway.	[15]
Avoid selecting designs in exon 1.	A possible alternative first exon use will be avoided. In addition, first exons generally have a higher methylation rate, possibly interfering with sgRNA binding.	[16,17]
Select the designs with the highest GC percentage, but not higher than 80%.	High GC percentages were shown to result in higher indel rates.	[15,18]
Preferably select designs with G but not A directly upstream of PAM.	Accounting for the base pair directly upstream of PAM was shown to lead to higher indel rates.	[18]
Preferably select target sites in different exons and protein domains.	By selecting target sites in different regions of the gene, the chance of introducing indel mutations in crucial regions of the gene is increased (in case this is not completely known).	

Supplementary Table 3: gBlock designs for *slc2a10*, *pls3*, *tapt1a*, *myt1la* and *tprkb*

Gene	Design	exon	strand	protospacer + PAM	G-block sequence
slc2a10	NA	2	+	AAAGCAAAGATAACATGCGGAGG	CCGCTAGCTAATACGACTCACTATAGGAGCAAAGATAACATG<br>CGGGTTTTAGAGCTAGAAATAGCAAGTTAAAATAAGGCTAGT<br>CCGTTATCAACTTGAAAAAGTGGCACCGAGTCGGTGCTTTT
pls3	NA	7	+	GGCTGTCACGTGGTCAACATTGG	CCGCTAGCTAATACGACTCACTATAGGCTGTCACGTGGTCAA<br>CATGTTTTAGAGCTAGAAATAGCAAGTTAAAATAAGGCTAGT<br>CCGTTATCAACTTGAAAAAGTGGCACCGAGTCGGTGCTTTT
tapt1a	NA	4	+	ATGATGTACCATCTGATCCGAGG	CCGCTAGCTAATACGACTCACTATAGGGATGTACCATCTGAT<br>CCGGTTTTAGAGCTAGAAATAGCAAGTTAAAATAAGGCTAGT<br>CCGTTATCAACTTGAAAAAGTGGCACCGAGTCGGTGCTTTT
myt1la	NA	8	-	CCCCGCTACGACAGCTGCCAGAT	CCGCTAGCTAATACGACTCACTATAGGCTGGCAGCTGTCGTA<br>GCGGTTTTAGAGCTAGAAATAGCAAGTTAAAATAAGGCTAGT<br>CCGTTATCAACTTGAAAAAGTGGCACCGAGTCGGTGCTTTT
tprkb	1	2	+	TGTGGATGCATTCCAGATCCTGG	CCGCTAGCTAATACGACTCACTATAGGTGGATGCATTCCAGA<br>TCCGTTTTAGAGCTAGAAATAGCAAGTTAAAATAAGGCTAGT<br>CCGTTATCAACTTGAAAAAGTGGCACCGAGTCGGTGCTTTT
tprkb	2	2	+	GGATGCATTCCAGATCCTGGTGG	CCGCTAGCTAATACGACTCACTATAGGATGCATTCCAGATCCT<br>GGGTTTTAGAGCTAGAAATAGCAAGTTAAAATAAGGCTAGTC<br>CGTTATCAACTTGAAAAAGTGGCACCGAGTCGGTGCTTTT

Supplementary Table 4: PCR primers. If possible, primers should be designed in a way that PCR products larger than 300 bp are obtained, when applying the Nextera XT library preparation (Illumina, San Diego, CA).

Gene	Design	Primer sequence	
slc2a10	NA	F	TCACGGTTGGCATCTTGATA
		R	AAGTGCATCGTTTCGTTGTG
pls3	NA	F	ACCCTGTTTGTTTTCCCGTA
		R	TTGTCATTACCATGCCCAAG
tapt1a	NA	F	GCTTCTTTACATTGCGGTGTT
		R	CACAGCGTCATCAAAATTCG
myt1la	NA	F	GTTCGAGAAACGGTGGACTC
		R	TGCCATTCTGTCTCGCATAA
tprkb	1	F	CCGTGTATCTCCTCACTTGC
		R	CAACATAGGGAGAACATGCAAA
tprkb	2	F	TCACCCCAAGCACATAAACA
		R	CGCCAACATAGGGAGAACAT

Installation notes

To install BATCH-GE, open a terminal on a Linux system and follow the underneath instructions:

1. Enter "git clone https://github.com/WouterSteyaert/BATCH-GE.git"
   This will clone the Git repository on your system. Make sure Git is installed on your system. If not, enter 'sudo apt-get install git'.
2. Change directory to BATCH-GE by entering 'cd BATCH-GE'
3. If your system has a web accessible folder in which you have write access enter 'perl Install.pl --WebAccFolder=LOCATION WEB ACCESSABLE FOLDER' and replace LOCATION WEB ACCESSABLE FOLDER by the full path to this directory. If your system hasn't any web accessible folder, simple run 'perl Install.pl'. The consequence will be that the URLs generated by BATCH-GE won't work. It has no consequences to the rest of the script.

The software needs a reference sequence to map the reads onto. This reference sequence must be located in the subfolder 'genomes' of BATCH-GE. Follow the underneath instructions to download and prepare the danRer7 genome in order to be able to run the examples. Other genomes can be downloaded and prepared in exactly the same manner. These steps only need to be performed once for each reference sequence.

1. Change directory to the subfolder 'genomes' by entering 'cd genomes'
2. Download the appropriate genome, for danRer7 enter 'wget ftp://hgdownload.cse.ucsc.edu/goldenPath/danRer7/bigZips/danRer7.fa.gz'
   This will download danRer7 from the UCSC ftp site.
3. Change directory to BATCH-GE by entering 'cd ..'
4. Prepare the genome for analysis by entering 'perl PrepareGenome.pl --Genome=danRer7'.
   This script will create a sequence dictionary and an index necessary in the further analysis.

Test the software by entering 'perl BATCH-GE.pl --ExperimentFile PREFIX/BATCH-GE/_EXAMPLE_1/ExperimentFile.csv'. Replace PREFIX by the folder in which you have cloned BATCH-GE. The mutagenesis efficiency is 0.11 and the efficiency of HDR is 0.01. Full results can be found in PREFIX/BATCH-GE-Pub/_EXAMPLE_1/Output/.

Replace _EXAMPLE_1 by _EXAMPLE_2 to run the second example. The mutagenesis in this example is 0.17 for sample 52 and 0.22 for sample 53. The HDR efficiency is 0.06 and 0.05 respectively. Full results can be found in PREFIX/BATCH-GE-Pub/_EXAMPLE_2/Output/

References

1 Fu, Y. F. *et al.* High-frequency off-target mutagenesis induced by CRISPR-Cas nucleases in human cells. *Nat Biotechnol* **31**, 822-+, doi:10.1038/nbt.2623 (2013).

2 Hsu, P. D. *et al.* DNA targeting specificity of RNA-guided Cas9 nucleases. *Nat Biotechnol* **31**, 827-+, doi:10.1038/nbt.2647 (2013).

3 Hwang, W. Y. *et al.* Efficient genome editing in zebrafish using a CRISPR-Cas system. *Nat Biotechnol* **31**, 227-229, doi:Doi 10.1038/Nbt.2501 (2013).

4 Li, M. H. *et al.* Efficient and Heritable Gene Targeting in Tilapia by CRISPR/Cas9. *Genetics* **197**, 591-U219, doi:10.1534/genetics.114.163667 (2014).

5 Kim, S., Kim, D., Cho, S. W., Kim, J. & Kim, J. S. Highly efficient RNA-guided genome editing in human cells via delivery of purified Cas9 ribonucleoproteins. *Genome research* **24**, 1012-1019, doi:10.1101/gr.171322.113 (2014).

6 Sung, Y. H. *et al.* Highly efficient gene knockout in mice and zebrafish with RNA-guided endonucleases. *Genome research* **24**, 125-131, doi:10.1101/gr.163394.113 (2014).

7 Irion, U., Krauss, J. & Nusslein-Volhard, C. Precise and efficient genome editing in zebrafish using the CRISPR/Cas9 system. *Development* **141**, 4827-4830, doi:Doi 10.1242/Dev.115584 (2014).

8 Hruscha, A. *et al.* Efficient CRISPR/Cas9 genome editing with low off-target effects in zebrafish. *Development* **140**, 4982-4987, doi:10.1242/dev.099085 (2013).

9 Hwang, W. Y. *et al.* Heritable and Precise Zebrafish Genome Editing Using a CRISPR-Cas System. *Plos One* **8**, doi:ARTN e68708 10.1371/journal.pone.0068708 (2013).

10 Wu, X. B. *et al.* Genome-wide binding of the CRISPR endonuclease Cas9 in mammalian cells. *Nat Biotechnol* **32**, 670-+, doi:10.1038/nbt.2889 (2014).

11 Jiang, W. Y., Bikard, D., Cox, D., Zhang, F. & Marraffini, L. A. RNA-guided editing of bacterial genomes using CRISPR-Cas systems. *Nat Biotechnol* **31**, 233-239, doi:10.1038/nbt.2508 (2013).

12 Jao, L. E., Wente, S. R. & Chen, W. B. Efficient multiplex biallelic zebrafish genome editing using a CRISPR nuclease system. *P Natl Acad Sci USA* **110**, 13904-13909, doi:10.1073/pnas.1308335110 (2013).

13 Ansai, S. & Kinoshita, M. Targeted mutagenesis using CRISPR/Cas system in medaka. *Biol Open* **3**, 362-371, doi:10.1242/bio.20148177 (2014).

14 Cong, L. *et al.* Multiplex Genome Engineering Using CRISPR/Cas Systems. *Science* **339**, 819-823, doi:10.1126/science.1231143 (2013).

15 Wang, T., Wei, J. J., Sabatini, D. M. & Lander, E. S. Genetic Screens in Human Cells Using the CRISPR-Cas9 System. *Science* **343**, 80-84, doi:10.1126/science.1246981 (2014).

16 Brenet, F. *et al.* DNA Methylation of the First Exon Is Tightly Linked to Transcriptional Silencing. *Plos One* **6**, doi:ARTN e14524 doi:DOI 10.1371/journal.pone.0014524 (2011).

17 Kimura, K. *et al.* Diversification of transcriptional modulation: Large-scale identification and characterization of putative alternative promoters of human genes. *Genome research* **16**, 55-65, doi:10.1101/gr.4039406 (2006).

18 Gagon, J. A., Valen, E., Thyme, S. B., Huang, P. & Ahkmetova, L. Efficient Mutagenesis by Cas9 Protein-Mediated Oligonucleotide Insertion and Large-Scale Assessment of Single-Guide RNAs (vol 9, e98186, 2014). *Plos One* **9**, doi:ARTN e106396 10.1371/journal.pone.0106396 (2014).
